# Comparative Analysis of the Effects of Acidic and Alkaline Beverages on the Optical Properties, Surface Topography, and Bacterial Activity of Zirconia Materials

**DOI:** 10.3390/jfb16090329

**Published:** 2025-09-05

**Authors:** Nasser M. Alahmari

**Affiliations:** Prosthetic Department, College of Dentistry, King Khalid University, Abha 62529, Saudi Arabia; nmr.dnt@gmail.com

**Keywords:** zirconia, color stability, mean color changes, surface topography, *Streptococcus mutans*, smokeless tobacco, alkaline media, acidic media

## Abstract

This study aimed to evaluate how acidic and alkaline staining solutions affect the optical properties (mean color change, ΔE*), geometric characteristics (surface roughness, Ra), and bacterial adhesion of zirconia Ceramill Zolid PS computer-aided design/computer-aided manufacture (CAD/CAM) material after 21 days of immersion. Ninety-six zirconia CAD/CAM Ceramill Zolid multilayer PS specimens were prepared and allocated to eight groups based on the pH values of the immersion solutions; the acidic solutions included Mirinda Citrus, CodeRed, yerba mate tea, Saudi coffee, and Nescafe (A–E), and the alkaline solutions included artificial saliva, DZRT (tobacco-free nicotine pouches), and smokeless tobacco (F–H). The specimens were immersed for 21 days at 37 °C, with the solutions replaced every 12 h to ensure consistency. Color changes were measured using a VITA Easyshade V spectrophotometer, and Ra was evaluated via white-light interferometric microscopy. The bacterial adhesion of *Streptococcus mutans* was quantified by counting colony-forming units (CFUs, CFU/mm^2^). Statistical analyses included the Shapiro–Wilk test for normality, one-way ANOVA with Tukey’s HSD post hoc test for group comparisons, and paired t-tests, with significance set at <0.05. The recorded pH values of the staining materials ranged from acidic (Mirinda Citrus: 3.23) to alkaline (smokeless tobacco: 8.54). Smokeless tobacco caused the most unacceptable mean color change (ΔE* = 6.84), followed by DZRT (ΔE* = 6.46), whereas artificial saliva produced the least discoloration (ΔE* = 2.15), with statistically significant differences among the solutions (*p* < 0.001). The Ra measurements varied significantly (*p* < 0.001), with Nescafe demonstrating the lowest value (0.486 µm) and DZRT the highest (0.748 µm). *S. mutans* adhesion was the highest for CodeRed (546.75 CFU) and the lowest for smokeless tobacco (283.92 CFU), demonstrating significant variation across groups (ANOVA, *p* < 0.001). The acidic and alkaline solutions significantly altered the optical properties, Ra, and bacterial adhesion of zirconia Ceramill Zolid PS CAD/CAM, with acidic solutions leading to higher bacterial adhesion.

## 1. Introduction

Zirconia-based dental restorations encounter significant mechanical and chemical challenges in the oral environment [1]. Mastication forces subject these restorations to cyclic loading and thermal fluctuations, which can induce microstructural defects such as microcracks and surface wear [1,2]. Surface degradation and wear are intensified by frequent consumption of acidic or alkaline beverages, which compromise the ceramic’s optical characteristics [3,4]. Pigmented compounds in such drinks penetrate the restoration’s microscopic pores, resulting in visible staining, while chemical erosion roughens the surface [5].

Increased surface roughness (Ra) facilitates colonization by cariogenic bacteria, including *Streptococcus mutans* and *Lactobacillus* species, promoting biofilm growth. Persistent biofilm accumulation contributes to plaque formation, secondary caries, and degradation at the restoration–tooth interface [6]. This cumulative damage accelerates fatigue failure, ultimately shortening the clinical lifespan of zirconia restorations.

The oral environment subjects ceramics to dynamic pH shifts, ranging from acidic (pH~2.0) during consumption of citrus beverages to alkaline under certain conditions [7]. These fluctuations in pH can degrade zirconia’s mechanical properties by reducing flexural strength, hardness, and fracture toughness [7,8]. This degradation is linked to yttria loss at low pH, making zirconia more prone to phase transformation from tetragonal to monoclinic, which has inferior mechanical properties [8]. Although aging progresses slowly at oral temperatures, factors such as thermal fluctuations and cyclic masticatory loads can accelerate degradation. High temperatures can exacerbate the aging process, increasing the monoclinic phase and degrading the mechanical properties [9]. The surface degradation and wear of zirconia restorations in acidic oral environments depend on the acid type, concentration, pH, exposure duration, and temperature [10,11]. Low-pH, high-viscosity lubricants prolong contact time, intensifying surface deterioration and opposing tooth wear [12].

The CIE (Commission Internationale de l’Éclairage) L*a*b* color system provides a standardized method for quantifying color changes [13]. In this system, (1) L* denotes brightness, (2) a* indicates red–green chroma, and (3) b* represents yellow–blue chroma. However, interpreting these values individually can be complex and potentially misleading [14]. Instead, researchers often rely on the ΔE: CIELAB (ΔELab) formula for determining mean color difference, which provides a numerical metric that captures even subtle variations in color perception [11]. This method is commonly used in dentistry to evaluate the color parameters as L, a, and b and the mean color changes (ΔE*) of dental materials [15,16,17]. The clinically acceptable ΔE* values are between 1.7 and 3.3 [18].

A change in the color of zirconia restorations is considered to be a treatment failure, especially when the esthetic region is involved [19]. Studies have shown that exposure to staining agents, such as carbonated beverages and tobacco extracts, can significantly alter the color parameters of zirconia [17]. Immersion in these substances leads to a decrease in the L* value, indicating darkening, and an increase in the a* and b* values, resulting in a shift toward red and yellow hues [20]. Discoloration of prosthetic materials is influenced by factors such as immersion time and surface treatment [21]. Glazed Ceramill Zolid PS exhibits an acceptable ΔE* 00 (1.58) after staining exposure [22]. Moaleem et al. (2021) found that Ceramill Zolid PS samples demonstrated lower ΔE* values than other materials after prolonged immersion in Coca-Cola [23]. Various social habits (e.g., khat chewing, smokeless tobacco, and coffee/fruit juice consumption) significantly influence the optical properties of lithium disilicate and zirconia restorations [15,24].

The surface characteristics, morphology, and bacterial adhesion resistance of dental materials are critical factors in determining the extent of bacterial colonization [25]. Ceramic restorative materials differ in texture and physicochemical traits such as hydrophobicity, surface energy, and chemical composition, which directly influence bacterial adhesion [26]. Rough surfaces contribute to discoloration and promote bacterial retention, increasing the risk of biofilm formation, particularly when roughness exceeds > 0.2 µm [27]. Glazing significantly reduces the Ra of acidic beverages, thereby enhancing the clinical performance and longevity of restorations [28]. Studies have consistently demonstrated that glazed ceramic surfaces exhibit increased color stability, decreased roughness alteration, and bacterial colonization compared with unglazed or inadequately polished surfaces [29,30]. Immersion of various restorations in khat extract can induce microporosities, thereby exacerbating Ra, with Ceramill Zolid PS zirconia exhibiting lower roughness than other CAD/CAM materials [22].

*S. mutans* has been recognized as one of the major etiological mediators of the initiation of dental caries lesions and/or secondary caries with microbial infection, which concerns most dentists and results in prosthetic treatment failure [31]. Once the cemented restoration sides are exposed to oral dentition, they are promptly colonized by microbes [32]. Multilayer zirconia as a prosthetic material exhibits the lowest values for the adhesion of bacteria [33]. Vulović reported that polished zirconia specimens accumulated fewer bacteria than glazed zirconia [34]. Other authors reported that the composition of biofilms formed on the surfaces of dental restorations is not only material-dependent but also individual-specific, with frequent sugary drink intake being associated with higher accumulation of bacteria [35].

Previous studies have investigated the effects of various media with different pH values on the mean color changes [15,23,28] or the alterations in surface morphology [23,24,28,29]. To the best of the author’s knowledge, no laboratory studies have been conducted on the mean color changes, surface topography alterations, and bacterial colony-forming units (CFUs) after staining caused by acidic and alkaline beverages. The present study aimed to assess and measure the ΔE* and surface topography alterations and to count the CFUs of Ceramill Zolid PS zirconia material following 21 days of staining in different consumed acidic media (including Mirinda Citrus, CodeRed, yerba mate, Saudi coffee, and Nescafe) and alkaline media (including artificial saliva, DZRT (snuff), and smokeless tobacco). The null hypothesis was that the ΔE*, surface topography alteration values, and CFU numbers after 21 days of staining by different acidic and alkaline media would show no significant differences.

## 2. Materials and Methods

### 2.1. Study Design and Sample Size Calculation

This study was designed to assess the mean color change (ΔE*), surface topography, and *S. mutans* adhesion (CFU) of zirconia CAD/CAM ceramics after 21 days of staining by acidic and alkaline media. The sample number was calculated using G*Power software (version 3.1.9.4, University of Dusseldorf). The effect size (d) was 0.5, the significance level (α) was 0.05, and 1-β (power) was 0.87. The required sample size was 96 samples divided equally among 8 staining media groups for detecting a large effect size (0.40) with a 1-β (power) of 0.80.

### 2.2. Specimen Manufacturing

The total 96 specimens were prepared from the zirconia CAD/CAM prosthetic material—Ceramill Zolid multilayer PS (Amann Girrbach, Pforzheim, Germany). The dimensions of the round zirconia discs were standardized, with a uniform diameter of 6.00 ± 0.25 mm and a thickness of 2.00 ± 0.25 mm. The discs were constructed in accordance with the manufacturer’s instructions using a CAD/CAM machine (Amann Girrbach, Pforzheim, Germany). The glazing process involved applying a GC glaze material onto the zirconia discs’ surfaces and subjecting them to a firing cycle according to the manufacturer’s instructions.

### 2.3. pH Measurement

Each of the acidic and alkaline staining media was prepared in accordance with its actual use during the immersion states of the zirconia samples to measure the pH. Then, the samples were placed inside the experimental glass test tube of the pH device (PHS-1705 Benchtop pH Meter; Shanghai BOQU Instrument Co., Ltd., Shanghai, China). A pen was introduced, and a numerical screen displayed the pH values and readings of each acidic and alkaline medium [36,37]. These procedures were repeated for all of the acidic and alkaline staining groups. For each immersion medium, three readings were recorded, and the average was considered to be the pH of the staining and immersing material.

### 2.4. Sample Grouping

The specimens were divided into eight groups (n = 12) in accordance with the pH of the media: Mirinda Citrus, CodeRed, yerba mate tea, Saudi coffee, and Nescafe as acidic media, and artificial saliva, DZRT (snuff), and smokeless tobacco as alkaline media.

### 2.5. Color Parameter Measurements

Before the color measurements, all of the CAD/CAM zirconia discs were cleaned by rinsing under deionized water for 30 s, ultrasonic bathing in deionized water (5 min) and 70% ethanol (5 min), and then rinsed again and dried with oil-free compressed air. The spectrometer was calibrated before use, and all of the discs were placed on a white background. Then, the first readings of the color parameters (baseline values) were recorded. L1, a1, and b1 refer to the color data measured before staining (baseline) with acidic and alkaline media, while L2, a2, and b2 refer to the color data following 21 days of immersion in the media.

The recorded values were utilized to calculate ΔE*, which is defined as the difference between two colors in an L*a*b* color space. The values were evaluated by calculating the difference in color measurements of the circular discs before and after 21 days of immersion in acidic and alkaline media, using the following formula [15,29]:

ΔE* = [(L1* − L2*)^2^ + (a1* − a2*)^2^ + (b1* − b2*)^2^]^1/2^

where L1 and L2, a1 and a2, and b1 and b2 refer to the values before and after immersion and staining, respectively. Then, ΔL*, Δa*, and Δb* were calculated. The chromatic values of the assessed circular CAD/CAM zirconia discs were measured on the center of each sample three times with the spectrometric device, and then the averages were documented and re-recorded for further measurements.

### 2.6. Staining of Glazed Zirconia Circular Discs

All of the glazed circular zirconia samples were immersed and stained in the acidic media Mirinda Citrus (A), CodeRed (B), yerba mate tea (C), Saudi coffee (D), and Nescafe (E). Mirinda Citrus and CodeRed were used in their ready-to-use commercial forms. An acceptable amount of each liquid was poured to acquire an adequate volume of 150 mL per solution for immersing the zirconia CAD/CAM samples. Meanwhile, yerba mate tea, Saudi coffee, and Nescafe were prepared in accordance with their manufacturers’ instructions; that is, 15 g of each staining material (powdered or packaged) was used for 250 mL of boiled distilled water (Berkshire, UK). Yerba mate tea staining solutions were added to 15 g–250 mL of boiling distilled water to achieve the homogeneity of the drink [15]. All staining powders and solutions were replaced daily and stirred once every 12 h to maintain freshness and homogeneity [15].

Similarly, the specimens were stained for 21 days in the alkaline media—artificial saliva (F), DZRT (G), and smokeless tobacco (H)—as described above. An adequate amount of artificial saliva was used to cover the samples in their containers. For DZRT and smokeless tobacco, each staining material was mixed with distal water to a thick and homogeneous consistency, and then the mixture was placed over the samples in the containers for 10 min, which corresponded to the typical duration of oral exposure during actual use. A constant weight was applied to each circular disc to ensure even immersion and simulate the intraoral compression produced during actual usage [16].

The specimens were immersed and stained in different acidic and alkaline media in separate glass containers; the media were shaken well before usage and altered every 12 h. All circular CAD/CAM zirconia discs immersed in the staining solutions were stored in an incubator at 37 °C during the 21-day staining period. The A–H specimen groups were cleaned using a regular soft toothbrush bristle with dentifrice (Signal^®^ toothpaste) for 20 s two times a day when changing the acidic and alkaline staining media, with a 2.0 ± 0.2 kg brushing force that was calibrated with standard masses prior to each session. After the staining period, the eight groups of specimens were removed from the staining media, and then the discs were dipped in distilled water 10 times, wiped with tissue paper, and left to air-dry completely before further assessment. One trained operator (N M A) performed all of the color measurements.

### 2.7. Surface Roughness Measurements

White-light interferometric microscopic images of the tested circular CAD/CAM zirconia specimens were taken after staining for 21 days in different acidic (A–E) and alkaline (F–H) media. The red and blue areas represent the part of the surface with the highest (i.e., the peaks) and lowest (i.e., the valleys) values, respectively. Three circular specimens from each acidic and alkaline group were scanned at three given points and averaged accordingly to determine the Ra value in μm. The Ra measurements followed the ISO 25178-2:2012 recommendations for standardization [16,38,39].

### 2.8. Bacterial Adhesion Analysis

The same number of samples was used for bacterial adhesion (*S. mutans*) incubation. After acidic or alkaline staining for 21 days, bacterial adhesion was assessed. A standardized bacterial suspension of *S. mutans* (approximately 1 × 10^8^ CFU/mL, adjusted by optical density) was prepared in brain heart infusion (BHI) broth supplemented with 1% sucrose. The specimens were incubated at 37 °C in 10% CO_2_ for 24 h with 1 mL of this bacterial suspension. After incubation, the specimens were rinsed with phosphate-buffered saline (PBS) to remove non-adherent bacteria. Adherent bacteria were collected by swabbing the surface with a sterile cotton applicator and suspending it in 100 μL of PBS. The suspension was then serially diluted and plated on Mitis Salivarius Agar (SDA) plates. Colony-forming units (CFUs) were counted manually using a colony counter (Fisher Scientific, CL334, Hampton, NH, USA), following established protocols [31,40]. The results were expressed as CFU/mm^2^.

### 2.9. SEM for Surface Topography Assessment

After 21 days of staining with acidic or alkaline media, zirconia samples (n = 8) were randomly selected from each group to analyze their surface topography by utilizing SEM (FEG 250-FeiQuanta, Eindhoven, The Netherlands). For each group, the most representative images were selected and archived for illustration and imaging. Each sample was scanned, and images were captured at 20 kV and 500× magnification.

**Figure 1:**
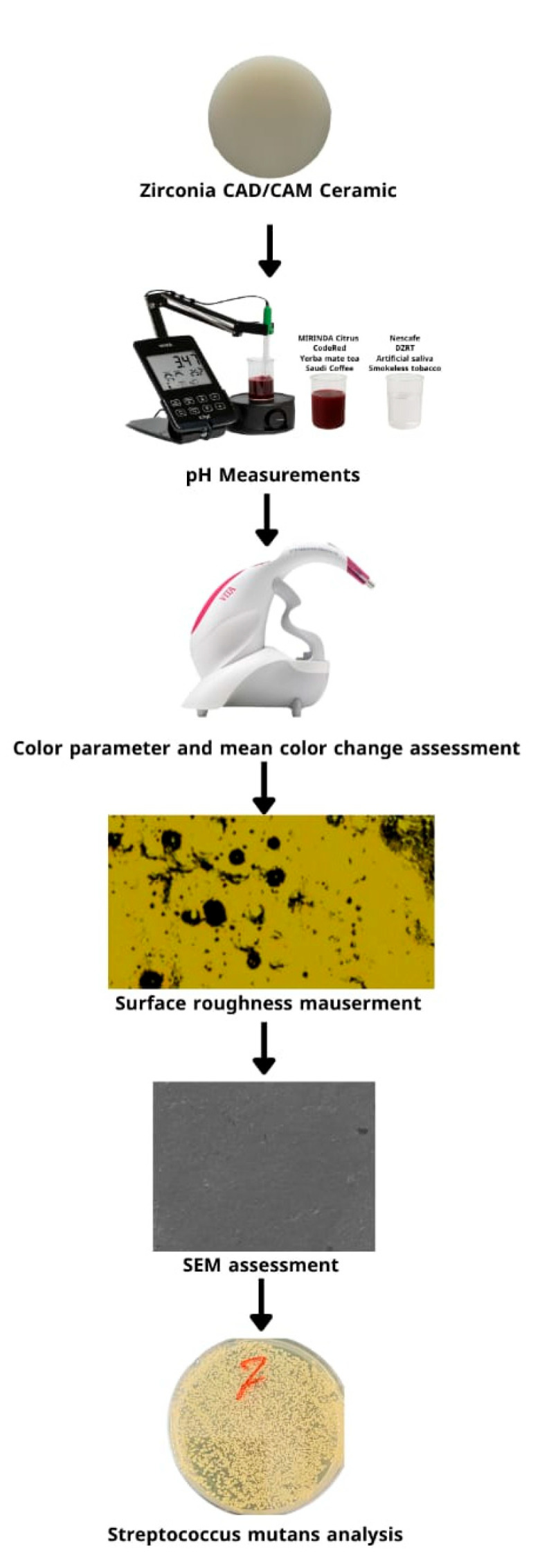
Study flowchart.

Table 1 shows the characteristics of the different materials and devices used in this in vitro study. Figure 1 displays the study flowchart, illustrating the mean color change, surface topography, *S. mutans* analysis, and SEM assessments.

### 2.10. Statistical Analysis

Data were analyzed using SPSS (version 26). Most of the data were normally distributed according to the Shapiro–Wilk test (*p* value ≥ 0.05). The mean and standard deviation were calculated for all variables. One-way ANOVA was utilized to examine significant differences in ΔE*, surface topography (Ra), and bacterial adhesion (CFU/mm^2^) values among the specimens immersed in different acidic and alkaline media. Post hoc tests using Tukey’s HSD were used for pairwise comparisons to identify differences across the immersion media. A *p* value < 0.05 was considered statistically significant.

## 3. Results

### 3.1. pH Values

The staining media, listed from the lowest to the highest pH, were as follows: Mirinda Citrus (A, 3.23), CodeRed (B, 3.34), yerba mate tea (C, 5.27), Saudi coffee (D, 5.64), and Nescafe (E, 5.69) for acidic media, and artificial saliva (F, 7.23), DZRT (G, 7.42), and smokeless tobacco (H, 8.54) for alkaline media.

### 3.2. Mean Values of the Color Parameter Changes

Table 2 presents the mean of the color parameters (ΔL, Δa, and Δb) of the materials after 21 days of immersion in acidic and alkaline staining media. The samples stained with Nescafe exhibited the most significant decrease in ΔL (−2.147), followed closely by those stained with Saudi coffee (−2.113), whereas the samples stained with DZRT showed the lowest change (−0.154). All of the tested media increased the Δa, with Nescafe resulting in the highest increase (0.743). The changes in Δb varied: the samples stained with CodeRed (−1.932) and Nescafe (−2.012) experienced decreases, whereas those stained with smokeless tobacco displayed the highest increase (3.652). Notably, DZRT uniquely increased the Δb (2.653), contrasting with other beverages such as Mirinda Citrus and yerba mate tea, which reduced the ΔL and Δb. Artificial saliva slightly increased the ΔL (0.103) but led to a decrease in Δb* (−1.212).

### 3.3. Mean Color Change, Surface Roughness, and Bacterial Adhesion

The highest ΔE* values were observed in the following order: smokeless tobacco ˃ DZRT ˃ CodeRed ˃ Nescafe ˃ Mirinda Citrus (6.84, 6.46, 4.47, 4.76, and 4.56, respectively). The lowest ΔE* was observed in artificial saliva. The ANOVA test indicated significant differences in the mean ΔE* values across different acidic and alkaline staining solutions (*p* < 0.001, Table 3).

Also, Table 3 shows that the highest mean Ra values were obtained in the following order: DZRT ˃ Saudi coffee ˃ Mirinda Citrus ˃ yerba mate tea ˃ smokeless tobacco (0.748, 0.587, 0.566, 0.514, and 0.505 µm, respectively). Meanwhile, the lowest mean Ra was observed for the samples stained with Nescafe (0.486 µm). The ANOVA test indicated significant differences in the mean Ra across different staining solutions (*p* < 0.001).

After 12 h of incubation, the samples stained with CodeRed exhibited the highest mean *S. mutans* adhesion (CFU/mm^2^) of 546.75, followed by the samples stained with Mirinda Citrus (510.92) and yerba mate tea (409.00). Meanwhile, the samples stained with smokeless tobacco showed the lowest mean CFU of 283.92. The ANOVA test indicated significant differences in the mean *S. mutans* adhesion after 12 h of immersion in acidic and alkaline staining solutions (*p* < 0.001) (Table 3).

Post hoc tests using Tukey’s HSD for pairwise comparisons of ΔE* showed that the samples stained with DZRT exhibited statistically significant differences from the samples stained with yerba mate tea, Saudi coffee, and artificial saliva (*p* = 0.001, 0.002, and <0.001, respectively). Similarly, the samples stained with smokeless tobacco displayed a significantly higher ΔE* than those stained with yerba mate tea, Saudi coffee, and artificial saliva (*p* = 0.004, 0.010, and <0.001, respectively). CodeRed showed a statistically significant difference when compared to artificial saliva (*p* = 0.031). Other comparisons, such as DZRT versus Mirinda Citrus (*p* = 0.073) and smokeless tobacco versus DZRT (*p* = 1.000), did not reach statistical significance (Table 4).

The results of the post hoc tests using Tukey’s HSD for pairwise comparisons showed that the samples stained with DZRT exhibited a significantly lower mean Ra than the samples stained with yerba mate tea, Saudi coffee, Mirinda Citrus, CodeRed, Nescafe, and artificial saliva (control), with differences of 0.234, 0.161, 0.182, 0.258, 0.262, and 0.245, respectively (*p* < 0.001 for all staining media). However, the samples stained with DZRT showed a higher Ra than the samples stained with smokeless tobacco, with a difference of 0.243 (*p* < 0.001), as shown in Table 4.

**Figure 2:**
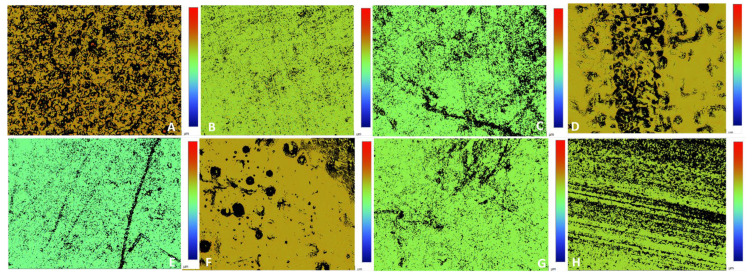
Ra images of glazed circular zirconia ceramics after 21 days of staining in acidic media—Mirinda Citrus (**A**), CodeRed (**B**), yerba mate tea (**C**), Saudi coffee (**D**), and Nescafe (**E**)—and alkaline media—DZRT (**F**), artificial saliva (**G**), and smokeless tobacco (**H**).

Table 4 also shows that staining with smokeless tobacco led to a significantly higher Ra than staining with Saudi coffee (difference of −0.082, *p* = 0.001) or Mirinda Citrus (difference of −0.062, *p* = 0.028). Meanwhile, staining with yerba mate tea resulted in a significantly lower Ra than staining with Saudi coffee (difference of −0.073, *p* = 0.004). Moreover, staining with Saudi coffee led to a significantly lower Ra than staining with CodeRed (difference of 0.097, *p* < 0.001) but a higher Ra than staining with Nescafe (difference of 0.101, *p* < 0.001) and artificial saliva (difference of 0.084, *p* = 0.001). Staining with Mirinda Citrus led to a significantly higher Ra than staining with CodeRed (difference of 0.077, *p* = 0.002), Nescafe (difference of 0.080, *p* = 0.001), and artificial saliva (difference of 0.063, *p* = 0.022). Figure 2 shows the Ra images of the glazed circular zirconia specimens after 21 days of staining with the acidic (A–E) and alkaline (F–H) staining media.

Post hoc tests using Tukey’s HSD for pairwise comparisons of the mean *S. mutans* after 12 h showed that the samples stained with DZRT exhibited a significant difference in bacterial adhesion compared with those stained with smokeless tobacco, Mirinda Citrus, CodeRed, and artificial saliva (*p* < 0.001 for all comparisons). Similarly, the samples stained with smokeless tobacco showed significant differences when compared with those stained with yerba mate tea, Saudi coffee, Mirinda Citrus, CodeRed, and Nescafe (*p* < 0.001 for all comparisons, Table 4).

**Figure 3:**
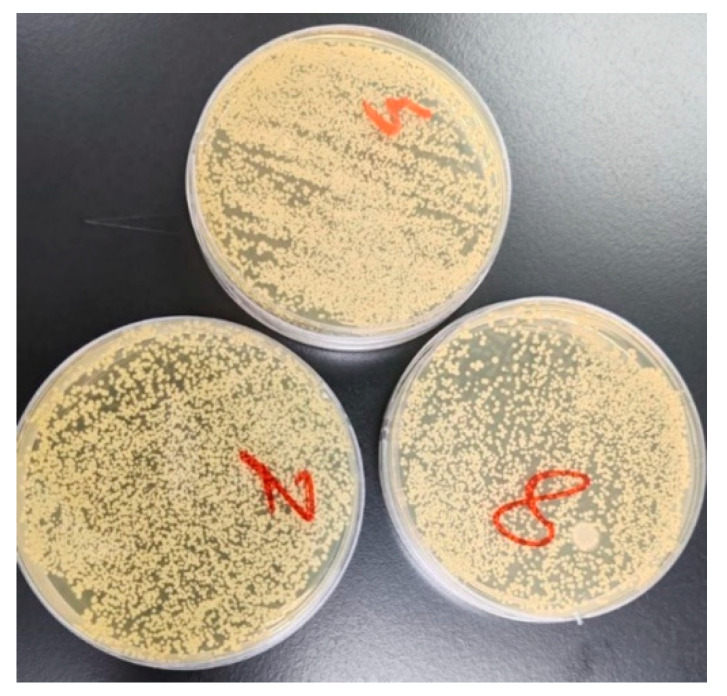
Bacterial adhesion after 24 h of incubation and 21 days of staining in an acidic medium—Nescafe (5)—and two alkaline media—artificial saliva (7) and smokeless tobacco (8).

Staining with acidic yerba mate tea demonstrated significant differences in bacterial adhesion when compared with Mirinda Citrus, CodeRed, and artificial saliva (*p* < 0.001). Samples stained with Saudi coffee showed significant differences from samples stained with Mirinda Citrus, CodeRed, and artificial saliva (*p* < 0.001). Samples stained with Mirinda Citrus exhibited significant differences from samples stained with Nescafe and artificial saliva (*p* < 0.001), but not from samples stained with CodeRed (*p* = 0.101). Staining with CodeRed led to significant differences compared with staining with Nescafe and artificial saliva (*p* < 0.001). Staining with Nescafe resulted in a significant difference compared with staining with artificial saliva (*p* < 0.001, Table 4). Figure 3 displays an image of *S. mutans* adhesion after 21 days of staining in the acidic medium Nescafe (5; group E) and the alkaline media artificial saliva (7; group G) and smokeless tobacco (8; group H).

### 3.4. Scanning Electron Microscopic Results

**Figure 4:**
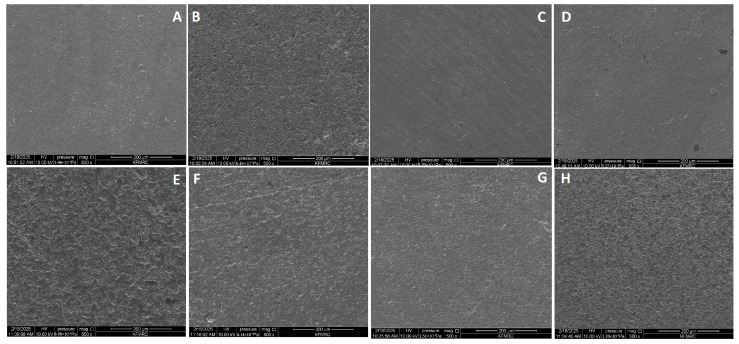
SEM images of tested glazed zirconia CAD/CAM ceramics at 500× magnification after 21 days of staining in acidic media—Mirinda Citrus (**A**), CodeRed (**B**), yerba mate tea (**C**), Saudi coffee (**D**), and Nescafe (**E**)—and alkaline media—DZRT (**F**), artificial saliva (**G**), and smokeless tobacco (**H**).

SEM revealed different morphological characteristics of the groups immersed in and stained with different acidic and alkaline solutions, as shown in Figure 4. All groups exhibited surface porosity as a common feature. However, the nature and distribution of these porosities differed between the acidic and alkaline groups, with the surfaces of the acidic groups stained with Mirinda Citrus, yerba mate tea, and Saudi coffee (Figure 4A,C,D) displaying a relatively homogeneous microstructural topography with randomly distributed pores. These pores were more visible on the surface of the DZRT group (Figure 4F). The control group, which was immersed in artificial saliva, maintained a relatively intact and smooth surface, representing a slight surface change.

## 4. Discussion

This study investigated how immersion in various acidic and alkaline solutions affects the color parameters, mean color changes, mechanical properties (surface alterations), and bacterial adhesion (measured in CFU) of Ceramill Zolid PS zirconia restorations. The immersion time was 21 days, corresponding to 36 months of clinical service [11].

The optical characteristics of teeth and dental restorations are evaluated on the basis of hue, value, and chroma [14]. In dentistry, the CIELab color system is widely used to measure these properties. This system defines three parameters: L* (lightness, where 0 is black and 100 is white), a* (representing the green–red axis, with negative values for green and positive for red), and b* (indicating the blue–yellow axis, with negative values for blue and positive for yellow) [13]. Previous studies have reported the high clinical service of zirconia restorations when exposed to acidic and alkaline media in the oral environment [41,42,43]. Although the CIEDE2000 formula provides superior perceptual uniformity and greater clinical relevance, we employed the CIELAB system in the present study to maintain methodological consistency and enable direct comparison with the previous literature [16,17].

This study found that the Δb* and ΔL* values of Ceramill Zolid PS zirconia restorations decreased after 21 days of immersion in most media, except for immersion in DZRT and smokeless tobacco, which showed an increase in Δb, shifting the hues toward yellow compared with the control group, where minimal changes occurred. These findings align with prior research that noted significant alterations in L, a, and b values following exposure to carbonated drinks, tobacco extracts [17,20], and acidic media [44].

The effects of patients’ daily acidic and alkaline beverage intake on the ΔE* of ceramic materials have been described in the literature. AlMoaleem et al. (2022), Alrabeah et al. (2023), Mugri et al. (2024), Alnasser et al. (2021), and Alnasser et al. (2019) [16,17,45,46,47] evaluated the effects of smokeless tobacco and electronic cigarettes, while other studies explored the effects of beverages with low pH values, such as Saudi coffee, khat, yerba mate tea, and Nescafe, on the color stability of prosthetic materials [15,48]. These studies found that the clinically acceptable color change values range from 1.7 to 3.7, with higher values considered to be marginally unacceptable. In the present study, all of the tested immersion beverages influenced the color of Ceramill Zolid PS zirconia, with overall values between 3.52 (±3.49) and 6.84 (±2.71), which are considered to be clinically acceptable, albeit marginally higher. These findings align with those reported by Alqahtani et al. for CAD/CAM ceramics exposed to traditional smoking (ΔE* > 3.3) and by Alrabeah et al. for monolithic zirconia exposed to electronic cigarettes [17,49]; they are also similar to the results recorded for zirconia materials after staining in acidic media [44].

The findings reject the first part of the null hypothesis, demonstrating that different staining agents significantly affect the ΔE* of Ceramill Zolid PS zirconia. Smokeless tobacco (ΔE* = 6.84) and DZRT (ΔE = 6.46) induced the most pronounced discoloration, exceeding clinically acceptable thresholds (ΔE* > 3.7) [16,18,46]. This effect likely stems from their unique chemical composition, particularly the presence of oxidizing metals (tin, nickel, and chromium) and viscous residues in their aerosols [45]. Previous research suggests that aerosols generated at higher power settings tend to exhibit stronger yellowish discoloration and enhanced adhesion, allowing for prolonged interaction with ceramic surfaces [17]. Despite zirconia’s inherent optical stability (3Y-TZP with residual cubic-phase content), these aerosols likely intensified light scattering and staining agent absorption, thus contributing to the pronounced ΔE* values [19].

Immersion in yerba mate tea, with a pH of 5.27, resulted in ΔL, Δa, and Δb values of −2.031, 0.621, and −1.731, respectively, which were different from the values recorded by Haralur et al. in 2019 [44], indicating ΔL, Δa, and Δb values of −0.918, 0.837, and 2.525 for monolithic zirconia and −0.259, 0.187, and −0.909 for bilayer zirconia, respectively. This finding could be explained by the type of tea used—with green tea being tested in the aforementioned study—and the composition and category of zirconia. Similarly, a ΔE* of 3.61 (±2.60) was obtained after 21 days of immersion in yerba mate tea. This value was higher than the value of 2.191 (2.18) recorded for monolithic zirconia, but much lower than that recorded for bilayer zirconia, which was 0.192 (2.18), and marginally similar to the value of 3.14 (1.60) recorded by Daghrery et al., 2024 [15]. The overall recorded ΔE* values for all of these studies were clinically acceptable.

The present study’s results are in contrast to the results of Zhang et al. (2021), who found minimal aging effects on zirconia’s color stability [50], and Prajapati et al. (2017), who reported no significant impact of smoking on various restorative materials [20]. Smokeless tobacco, which caused substantial discoloration in the present study, was associated with lower ΔE* values (2.7–4.8) in other CAD/CAM materials [16]. This difference can be attributed to the variations in material type and methodological differences, such as the exposure duration or the specific ceramic compositions tested.

Achieving color stability in dental materials depends on maintaining a microscopically smooth surface, because roughness can increase stain accumulation [29]. Ra is a critical factor in preserving surface integrity and preventing extrinsic discoloration [20,45]. According to the literature, the acceptable threshold for Ra is 0.2 µm, above which increased bacterial retention can be detected [26,27]. In the present study, all of the tested staining solutions resulted in Ra values exceeding this limit (≥0.486 µm), with significant differences among them, leading to the rejection of the second part of the null hypothesis. Different pH values promote the selective leaching of alkali ions, such as potassium and sodium, and can cause porosities, as observed on SEM photomicrographs [51]. The findings of the present study are in line with those of previous studies that reported deleterious effects of acidic agents on dental ceramics, as quantitatively and qualitatively tested using a profilometer and SEM images, respectively [39,49,52].

Bacterial adhesion (CFU/mm^2^) was notably greater in the acidic beverage groups, with the highest levels observed for CodeRed and Mirinda Citrus, leading to the rejection of the null hypothesis. This difference could be attributed to acidic drinks having a higher sugar content, which facilitates biofilm formation by providing nutrients for cariogenic bacteria [53]. Zirconia is generally considered to be hydrophobic, characterized by low surface wettability and reduced contact angles, properties typically associated with decreased bacterial accumulation [26]. However, the low pH of these beverages may contribute to chemical etching on ceramic surfaces, thus increasing the surface roughness [28]. These alterations in surface morphology enhance the conditions for microbial attachment.

In this study, the bacterial adhesion of *S. mutans* was high in all groups and much higher than that recorded in recent research [39,40]. This difference in findings could be attributed to the different pH values of the staining materials; previous research used alkaline media only (Saudi coffee), whereas the present study used both acidic and alkaline media, and the period of immersion was longer. Moreover, in terms of ceramic morphology, previous research tested full anatomical crowns, whereas the present study assessed flat discs. However, the CFU values recorded in the present study were much higher than those recorded in previous research using glazed or polished zirconia [33,34], which could have been due to the effect of 21-day staining using different materials.

Previous research suggests a possible relationship between surface topography and the color stability of dental restorations [54,55,56]. However, in the current study, no significant correlation was found between optical properties and Ra or bacterial adhesion for Ceramill Zolid PS zirconia, irrespective of the staining medium’s pH. This finding implies that while staining agents may independently affect optical characteristics and surface texture, these factors do not directly influence one another under the tested conditions. Notably, the pH of the staining media showed a direct relationship with Ra, as demonstrated by the irregularities observed in the SEM images. Specifically, immersion in more acidic solutions, such as Mirinda Citrus and CodeRed, resulted in higher Ra values and more pronounced textural degradation. These findings align with those of existing studies, demonstrating that acidic environments can chemically etch ceramic materials, increasing microporosity and Ra [28,29] and, thus, bacterial adhesion [40].

To the best of the author’s knowledge, this study is the first to comprehensively evaluate the optical–mechanical properties and bacterial adhesion of Ceramill Zolid PS zirconia restorations after immersion in staining solutions with varying pH levels. While some of the findings align with those of previous research, this study provides novel insights into how pH variations in commonly consumed substances impact zirconia ceramic prostheses. These results offer clinically relevant guidelines for material selection, particularly for patients with habits such as tobacco use or frequent consumption of acidic beverages, which can help in optimizing esthetic outcomes and long-term restoration performance.

This study has some inherent limitations. Despite rigorous methodologies, the controlled conditions could not fully replicate the dynamic oral environment, such as the effects of salivary flow, temperature variations, or mechanical stresses from chewing, which may alter material degradation and bacterial adhesion. While the 21-day immersion protocol provided short-term insights, it may not reflect long-term clinical outcomes over years of use. This study focused solely on *S. mutans* adhesion, overlooking the complexity of oral microbiomes, where polymicrobial interactions may yield different results. Additionally, only glazed zirconia Ceramill Zolid PS CAD/CAM was tested, and variations in surface treatments (e.g., polishing) or other restorative materials could produce distinct behavior. Furthermore, the fixed immersion protocols and sample size may limit the generalizability to real-world variability in dietary habits or staining agent exposure. Future studies should investigate the effect of materials’ surface wettability and incorporate in vivo simulations, extended timelines, and polymicrobial analyses to better mimic clinical conditions. We also suggest employing the CIEDE2000 (E00) formula in future studies to investigate color differences, considering reports in the literature about its superiority in terms of human perception of minor color variations [57,58].

## 5. Conclusions

This study found that immersion in acidic and alkaline solutions significantly affected the optical–mechanical properties and bacterial adhesion of Ceramill Zolid PS CAD/CAM restorations. Immersion in alkaline solutions, such as smokeless tobacco and DZRT, resulted in the highest ΔE* values. All solutions increased Ra, with Mirinda Citrus having the most significant impact. SEM revealed distinct porosity, with acidic solutions causing homogeneous degradation. Additionally, acidic solutions promoted greater bacterial adhesion (*S. mutans*) than alkaline solutions. No relationships among ΔE* values, Ra, and CFU were found for the acidic and alkaline solutions.

## Figures and Tables

**Table 1:** List of materials arranged according to their pH and devices used in this study.

Material/Device Type	Brand Name/pH	Composition/Description	Manufacturer	Color/Application Per Day
**Zircon CAD/CAM**	Ceramill Zolid multilayer PS	ZrO_2_ + HfO_2_ + Y_2_O_3_: ≥99.0, Y_2_O_3_: 8.5–9.5, HfO_2_: ≤5, Al_2_O_3_: ≤0.5, other oxides: ≤1	Amann Girrbach, Germany	B1 Light
**pH meter device**	PHS-1705 Benchtop pH Meter	Investigational glass test tube of the pH device with a pen and a digital screen displaying the values of pH	Shanghai BOQU Instrument Co., Ltd.	--
**Mirinda**	Mirinda Citrus carbonated soft drink/pH value of 3.23 (acidic)	Carbonated water, sugar, concentrated orange juice, citric acid, natural citrus flavor, caffeine, sodium benzoate (preservative), sodium citrate (acidity regulator), acacia gum (emulsifier), ascorbic acid, calcium-disodium EDTA (antioxidants), and color tartrazine (E102)	National Carbonated Beverages Co., Ltd. (PepsiCo KSA)	Bright yellow-green/two
**CodeRed**	Mountain Dew Code Red—cherry–citrus flavor/pH value of 3.34 (acidic)	Carbonated water, high-fructose corn syrup, concentrated orange juice, citric acid, sodium polyphosphates, sodium benzoate, natural flavor, caffeine, sodium citrate, Arabic gum, calcium-disodium EDTA, Red 40, Yellow 5, Blue 1	PepsiCo Beverages North America	Deep red/two
**Yerba mate tea**	ENVASADA ENORIGEN TARAGUI/pH value of 5.27 (acidic)	Caffeic acid, caffeine, caffeoyl derivatives, caffeoylshikimic acid, chlorogenic acid, feruloylquinic acid, kaempferol, quercetin, quinic acid, rutin, and theobromine	INDUSTERIA ARGENTEIN	Green/two
**Saudi Arabic coffee**	Saudi coffee mix (cardamom)/pH value of 5.64 (acidic)	Instant Saudi coffee, cardamom, cloves, nondaily coffee creamer, and saffron; used as hot coffee	Baja Food Industrial Co., Ltd. Jeddah, Saudi Arabia	Yellowish/two
**NESCAFE**	Nescafe, 3 in 1 (STRONG)/pH value of 5.96 (Acidic)	Sugar, glucose syrup, instant coffee (11%), palm kernel oil, soluble fiber, skimmed MILK powder (0.7%), MILK protein, salt, stabilizers, lactose (MILK), acidity regulator, emulsifiers, natural flavorings, MILK fat, and color	Nescafe, Saudi Arabia	Black/two
**Artificial synthesis saliva**	Unstimulated whole human saliva (pooled and filtered)/pH value of 5.27 (alkaline)	≈99% water; electrolytes Na^+^~80 mmol L^−1^, K^+^~8 mmol L^−1^, Cl^−^~40 mmol L^−1^, HCO_3_^−^ 15–25 mmol L^−1^, Ca^2+^~1–2 mmol L^−1^; proteins ≈ 0.5 g L^−1^ (amylase, mucins, proline-rich proteins, IgA, statherin); pH 6.2–7.6	Biochemazone^TM^ Arab Science Trading Co., Ltd. Riyadh, Saudi Arabia	Clear, slightly opalescent/two
**DZRT**	DZRT^®^ nicotine pouch, e.g., ‘Highland Berries 6 mg’/pH value of 7.42 (alkaline)	Cellulose plant fibers, water, pharma-grade nicotine 3/6/10 mg pouch^−1^, propylene glycol (humectant), sodium carbonate + bicarbonate (pH adjusters), acesulfame K or xylitol (sweeteners), food-grade flavorings, and trace NaCl	Badael International Co., Ltd. Riyadh, Saudi Arabia	White pouch/two (placed on specimens two times per day, for 30 min each time)
**Smokeless tobacco (Shamma)**	Black Shamma/pH value of 8.54 (alkaline)	Largely manufactured by powdering tobacco along with ash, flavors, oils, calcium oxide, and black pepper	Purchased in a plastic package from the market	Black/two
**Spectrophotometer**	VITA Easyshade Compact version V	Device used to measure wavelength transmitted from one object at a time, without being affected by subjective perceptions of color	VITA Zahnfabrik H. Rauter GmbH & Co. KG, Bad Sackingen, Germany	Measure the color parameters L, a, and b
**Surface roughness tester**	Profilometer	Device used to graphically record the average height of the profile above and below a center line along a given length of the sample	Perthometer M2, Mahr GmbH, Germany	Measure Ra
**Scanning electron microscope**	SEM	Device that uses a focused beam of electrons to scan the surface of a specimen and generate images at a much greater resolution than optical microscopy	East Sussex, BN8 6BN, UK	Measure the surfaces of samples
**Bacterial adhesion**	Bacterial colony-unit counting	Device for measuring *Streptococcus mutans* colony-forming units (CFU/mm^2^)	Fisher Scientific, CL334, NH, USA	Bacterial CFU/ mm^2^

**Table 2:** Mean ∆L, ∆a, and ∆b after 21 days of immersion in acidic and alkaline media.

Staining Medium	ΔL	Δa	Δb
	Mean	Mean	Mean
Mirinda Citrus	−1.846	0.563	−1.457
CodeRed	−1.948	0.643	−1.932
Yerba mate tea	−2.031	0.621	−1.731
Saudi coffee	−2.113	0.643	−1.598
Nescafe	−2.147	0.743	−2.012
DZRT	−0.154	0.642	2.653
Artificial saliva	0.103	0.432	−1.212
Smokeless tobacco	−0.234	0.546	3.652

**Table 3:** Mean color change (ΔE*), surface roughness (µm) after 21 days of immersion in acidic and alkaline media, and bacterial adhesion (CFU/mm^2^) after 12 h of incubation.

Staining Medium	Mean Color Change (±SD)	*p*-Value	Mean Surface Roughness (±SD)	*p*-Value	Mean Bacterial Adhesion (±SD)	*p*-Value
Mirinda Citrus	4.56 (±3.77)	<0.001 *	0.566 (±0.039)	<0.001	510.92 (±35.95)	<0.001
CodeRed	4.47 (±3.31)		0.489 (±0.025)		546.75 (±28.63)	
Yerba mate tea	3.61 (±2.60)		0.514 (±0.041)		409.00 (±29.27)	
Saudi coffee	3.52 (±3.49)		0.587 (±0.019)		390.83 (±17.27)	
Nescafe	4.76 (±1.52)		0.486 (±0.053)		382.58 (±37.18)	
DZRT	6.46 (±4.76)		0.748 (±0.092)		372.17 (±20.43)	
Artificial saliva (control)	2.15 (±2.66)		0.503 (±0.018)		306.33 (±37.34)	
Smokeless tobacco	6.84 (±2.71)		0.505 (±0.028)		283.92 (±35.29)	

ANOVA test; * indicates significant difference.

**Table 4:** Pairwise comparisons of mean color change (ΔE*), mean surface roughness (µm), and bacterial adhesion after 12 h of incubation between two staining media.

Pair of Staining Media	Mean Color Change (Unit)		Surface Roughness (µm)		Bacterial Adhesion (CFU/mm^2^)	
	Difference	*p*-Value	Difference	*p*-Value	Difference	*p*-Value
Mirinda Citrus vs. CodeRed	0.09	0.997	0.077	0.002	−35.83	0.101
Mirinda Citrus vs. yerba mate tea	0.95	0.815	−0.052	0.108	−101.92	<0.001 *
Mirinda Citrus vs. Saudi coffee	1.04	0.937	0.020	0.954	−120.08	<0.001 *
Mirinda Citrus vs. Nescafe	−0.20	1.000	0.080	0.001	128.33	<0.001 *
Mirinda Citrus vs. artificial saliva (control)	2.41	0.173	0.063	0.022	204.58	<0.001 *
Mirinda Citrus vs. DZRT	−1.90	0.073	0.182	<0.001	−138.75	<0.001 *
Mirinda Citrus vs. smokeless tobacco	−2.28	0.216	−0.062	0.028	−227.00	<0.001 *
CodeRed vs. yerba mate tea	0.86	0.382	0.025	0.882	−137.75	<0.001 *
CodeRed vs. Saudi coffee	0.95	0.582	0.097	<0.001	−155.92	<0.001 *
CodeRed vs. Nescafe	−0.29	0.999	0.003	1.000	164.17	<0.001 *
CodeRed vs. artificial saliva (control)	2.32	0.031	−0.014	0.996	240.42	<0.001 *
CodeRed vs. DZRT	−1.99	0.320	0.258	<0.001	−174.58	<0.001 *
CodeRed vs. smokeless tobacco	−2.37	0.623	0.015	0.991	−262.83	<0.001 *
Yerba mate tea vs. Saudi coffee	0.09	1.000	−0.073	0.004	18.17	0.839
Yerba mate tea vs. Nescafe	−1.15	0.732	0.028	0.802	26.42	0.433
Yerba mate tea vs. artificial saliva (control)	1.46	0.953	0.011	0.999	102.67	<0.001 *
Yerba mate tea vs. DZRT	−2.84	0.001	0.234	<0.001	−36.83	0.083
Yerba mate tea vs. smokeless tobacco	−3.23	0.004	−0.009	1.000	−125.08	<0.001 *
Saudi coffee vs. Nescafe	−1.24	0.888	0.101	<0.001	8.25	0.998
Saudi coffee vs. artificial saliva (control)	1.37	0.847	0.084	0.001	84.50	<0.001 *
Saudi coffee vs. DZRT	−3.12	0.002	0.161	<0.001	−18.67	0.819
Saudi coffee vs. smokeless tobacco	−3.32	0.010	−0.082	0.001	−106.92	<0.001 *
Nescafe vs. artificial saliva (control)	2.61	0.125	−0.017	0.985	76.25	<0.001 *
Nescafe vs. DZRT	−1.7	0.105	0.262	<0.001	−10.42	0.991
Nescafe vs. smokeless tobacco	−2.08	0.286	0.019	0.974	−98.67	<0.001 *
Artificial saliva vs. DZRT	4.31	<0.001	0.245	<0.001	65.83	<0.001 *
Artificial saliva vs. smokeless tobacco	−4.69	<0.001	0.002	1.000	−22.42	0.642
DZRT vs. smokeless tobacco	−0.38	1.000	0.243	<0.001	88.25	<0.001 *

Pairwise comparison; * indicates significant difference.

## Data Availability

The original contributions presented in this study are included in the article. Further inquiries can be directed to the author.

## Abbreviations

The following abbreviations are used in this manuscript:

ΔE*	Mean color change
Ra	Surface roughness
CFUs	Colony-forming units
*S. mutans*	*Streptococcus mutans*
